# Pathologia Generallis

**Published:** 1865-10

**Authors:** W. H. Price


### PATHOLOGIA GENERALLIS.

By W. H. PRICE.

Medicine may be compared to a great edifice, the foundation and chief entrances of which represent general pathology, which generally give the proper approach to the separate rooms, special diseases. Compare the state of the practice of medicine with that of anatomy, physiology and chemistry—the great fundamental or preparatory studies. How minute, how precise, how connected and definite are these, yet how loose—indefinite, uncertain, unconnected is the practice of medicine when not based upon its foundation, general pathology. To the public it appears altogether vague without any acknowled principles. Is there any wonder then that quackery should triumph, that the public show their want of faith in legitimate medicine by their ready belief in any novelty that is not legitimate. Thus one year St. John Long plan, another homœopathy, another Morrison's pills, another the water cure, rules the fashion. The public may show their ignorance by such credulity, but they show also the want of something plain and trustworthy in regular practice. The public will not believe that the secret of the art is with a faculty which professes to follow experience only. The quack also can appeal to his experience, and that too in a way more striking and convincing than those who express doubts and admit difficulties. Thus one who cures nervous diseases can calculate his success by the numerical method. In 8,000 cases he can count only 20 failures. Another will tell of an extraordinary percentage of success in cases of deafness, in which the most eminent practitioners had failed. Hence the partisans of quackery are more zealous in the defence of their favorite notions, than others are in support of the regular art. No wonder that homœopathy and the water cure have their royal and noble advocates. Then there is a captivating simplicity in the theories of quacks. A certain high official personage pins his faith to an empiric who was a gardener, and whose notion is, that all diseases proceed from buttercups. This is the theory, every man, woman and child eats mutton, beef, or butter, or drinks milk; every cow and sheep eats buttercups with its grass; buttercups are rank and acrid weeds; all diseases proceed from buttercups. How beautifully simple! How attractive, too, are the comprehensive views of the hygeist and the water curers! They both agree in their pathology; all diseases arise from bad matter in the blood; they only differ in their mode of expelling it from the system. One purges out the peccant humor, the other washes and sweats it forth.

On the other hand the regular practitioner has nothing so plausibie or so captivating to bring forward in explanation of his method, unless he has made general pathology the basis of his practice. He either has no theory at all and grounds his practice on experience, or if he gives a theory it is viewed only as an opinion, no better than the hypothesis of the quack in an art so little founded on principles as medicine, would be, without general pathology. General pathology is the basis of practical medicine, without it, it is impossible for the young practitioner to practice his art with any degree of success. To undertake the practice of medicine without a knowledge of general pathology, is like a person beginning the study of mechanics with the steam engine, or to the student of chemistry commencing with organic matter. The general result is, that where any distinct notion of disease is acquired, it is one not at all founded on previous physiological knowledge; but it is a new idea of disease as an absolute separate thing, not a mere condition consisting of altered function and structure, but a being, the character and history of which are to be detailed like that of a plant or an animal.

If now the student, who has not previously acquired any general ideas of pathology, but simply studied the dry mass of disease in detail, is followed to the bedside, well crammed with his nosological list and definitions, it will be seen how his knowledge, so meritoriously and laboriously obtained, will serve him in the hour of need. In a few cases of fully developed and well marked acute diseases, such as pleurisy, scarlet fever, or rheumatism, he may get on very well, but in the commoner description of cases, acute or chronic, in their early stages, in their endless variations, from peculiarities of constitution, or from complicating causes, he finds himself continually at a loss, the phenomena do not correspond with any of his defined diseases, they frequently change their character in a way that he cannot account for; his prognosis falsified, his diagnosis fails, and his treatment, although not always unsuccessful, does not answer according to his expectations—some patients recovering whom he expected to die, others dying or not improving whom he expected to recover. Disappointed in the failure of his nosological learning, the young practitioner more and more mistrusts it, and falls into a routine of empirical practice. Without troubling his head about the name or nature of diseases, he thinks solely of their treatment, and begrudging the time that he has spent with books and lectures, he decries everything that is not practical. Still he is obliged to retain some notions of the theory of disease; but they are general notions, and not fettered by definitions. He still studies symptoms, he seeks in the pulse and heat of skin indications of fever and inflammation; he looks to the tongue and alvine evacuations, for proofs of disorder of the digestive system; he judges by the complexion and muscular strength the state of the constitution. In short, he has in practice learned himself, in a loose way, at the expense of previous studies, and sometimes, it is to be feared, at the expense of some bad practice, what he ought to have previously studied as the foundation of his practice. This we are led to the conclusive presumption that general pathology is the proper basis for practical medicine, and I venture to affirm that the chief reason why the practice of medicine has been commonly so distasteful and so unsatisfactory when tested at the bedside, is because its foundation has not been efficiently studied.

I think that a knowledge of general pathology is a more efficient help at the bedside than such knowledge of diseases, as is to be obtained only from nosological definitions and details. Without the connecting link of general pathology, practical medicine derives little or no aid from anatomy and physiology. Instead of being founded upon them, it is studied and practised quite independent of a full knowledge of them, and is generally acquired in proportion as they are forgotten. This kind of practical medicine is much the same as that of old women and nurses, it consists chiefly of treating symptoms by remedies that have been found useful in similar cases, without the trouble of inquiring about the causes of the symptoms or the precise seat of the disease. Thus, if a person complains of headache and giddiness, leeches are applied, purgatives are given, because they have been found useful in similar cases. An intimate knowledge of the structure and functions of the contents of the head would give no farther help in the use of these remedies, nor suggest others, if these be found to fail. If they do fail, the only resource is in experiment: first one thing is tried, then another, until much mischief may be done, or at last, perchance, the right remedy may be hit upon, and this may be the very opposite of those first used. Long experience may make the symptom treating practitioner more successful, if he be an observing man, because it will acquaint him with additional symptoms to be considered for the guidance of the treatment. But there are few of this class of practitioners who are careful, observing men, and that profit by their experience. But the benefit of such experience is gained at the commencement by the student of general pathology. He has learned to trace symptoms to their causes. Having been taught by anatomy the peculiarities of the circulation in the head, and by physiology confirmed by clinical observation, that this circulation may be similarly impeded by opposite causes, inanition as well as fulness, he is prepared to find out through other symptoms which is the cause of the headache in the case before him, and he adapts his remedies accordingly. In fact, a true pathology and sound principles of medicine are the embodiment of the results of experience in disease, with a knowledge of structure and function in health.

General pathology is the only connecting link between the preparatory sciences and practical medicine. It needs only the growth and continued support of its chief members, especially anatomy, physiology and clinical observation, to become the perfect and efficient director of practical medicine.

The great proof of the practical utility of general pathology. is the aid which it gives in the study of clinical medicine, and the light which clinical medicine throws on it continually. The states which the practitioner has to treat are often too indefinite or too mixed to correspond with any of the definitions of special disease. They frequently consist of functional disorder, varying with time and circumstances, or changing its place so as to present no fixed characters. But compared by the pathologist with the standard of health and analyzed from their complexity, their nature becomes intelligible, and their proper treatment obvious so far as means are possessed to counteract or control that which is wrong. See in the disordered state of health, which some call disordered constitution, others indigestion, how the pathologist analyzes the symptoms of such a state, in the white or yellowish furred tongue, morbid crustations, tender epigastrium, sometimes full right hypochondrium, with extended dulness on percussion, the discolored fæces, the high colored and turbid urine. In these he finds proof of congestion and disturbed secretion of the liver and upper part of the alimentary canal. He recognizes in the remedies employed, means, which by increasing the secretions, relieve the congestion, and if these fail he can suggest other measures which he knows to be efficient in removing congestion and restoring the natural secretions. Again great confusion in diagnosis as well as in practice, has arisen from comprehending under the specific name of hysteria the most opposite and the most varying conditions, merely because they are consorted with some nervous phenomena, so that this word becomes almost synonymous with female diseases. But pathologically considered, the confusion in diagnosis, and in some measure the perplexity in regard to treatment, cease. In one case the pathologist finds the uterine function impaired, but this only in common with other functions. In this condition of the system he finds a want of blood throughout the body, which want is denoted by the waxy complexion, the pallid lips and gums, the loose yet easily quickened pulse, the panting breath, the feeble limbs, &c. Here the restoration of the blood is the obvious indication, and in proportion as this is effected, the symptoms of nervousness, debility and loss of function disappear. In another case called hysteria, the pathologist discovers the opposite condition, that of sanguineous plethora, which, independently of any disorder of the uterus, causes trouble in different parts of the economy, but especially in the nervous system, which in most females is peculiarly liable to disorder. Here, too, he is led to the most appropriate treatment. This is but one instance out of many that might be adduced to show the great practical utility of general pathology. In fact the leading rules, those which guide the most experienced men, are founded on general views of diseased function and structure. Practical men do not treat a disease merely according to its name or according to the nature of the local mischief. Inflammation is not always to be combated by venesection nor hemorrhage by styptics. The condition of the system, that is, of the functions, is to be duly considered, and the variations of this condition, the states of 'sthenia and and asthenia, tone and debility, excitement and depression, plethora and anæmia.

It is a fact, that practitioners do act more on general ideas of disease than on their knowledge of particular diseases. They feel the pulse and the skin to guide them in the use of venesection, whether they have found out the special disease or not. They examine the tongue and inquire as to the state of the evacuations to guide them in the use of purgatives, under whatever complaint the patient labors. They consider the complexion and bodily strength in connection with dietetic measures, and the chief treatment of convalescence depends on rules suggested by general pathological knowledge. It is then very important that these general views, which are so practical and so extensive in their application, should be well studied, and that the leading doctrines of disease should not be left to be picked up from casual retrospects of experience, but they should be learned as the very ground-work of practical knowledge. What, then, in this general pathology which is so much extoled by the profession as the proper foundation of practical medicine? It is not a collection of hypotheses hung on solitary facts, and ingeniously devised to explain this or that symptom, or the *modus-operandi* of this or that remedy. It is not anything floating on conjectural notions in anatomy and physiology, such as the existence and circulation of a nervous fluid, or the vital attractions and repulsions of the circulating fluids, notions which, however they may hereafter be substantiated, are at present too speculative to form a foundation for pathology. Nor is it a partial set of opinions erected on one only of the many pediments of fact on which the science of medicine should stand. Healthy anatomy, physiology, physics, chemistry, the study of clinical medicine, that of materia medica, morbid anatomy—neither of these alone can furnish a basis for pathology—that basis must be formed by ALL the facts which all supply, constitute the material of which it is built, and the general facts or laws of all must be brought to bear on the arrangement of these materials in the construction of a system of pathology. In fact general pathology is like the trunk and great branches of a tree, of which special pathology constitutes the leaves and boughs—all preserving an identity and connection, yet each portion having peculiarities of character which require seperate study.

